# Intelligently Targeted Drug Delivery and Enhanced Antitumor Effect by Gelatinase-Responsive Nanoparticles

**DOI:** 10.1371/journal.pone.0069643

**Published:** 2013-07-30

**Authors:** Rutian Li, Wei Wu, Qin Liu, Puyuan Wu, Li Xie, Zhenshu Zhu, Mi Yang, Xiaoping Qian, Yin Ding, Lixia Yu, Xiqun Jiang, Wenxian Guan, Baorui Liu

**Affiliations:** 1 The Comprehensive Cancer Center of Drum-Tower Hospital, Medical School of Nanjing University & Clinical Cancer Institute of Nanjing University, Nanjing, P.R. China; 2 Laboratory of Mesoscopic Chemistry and Department of Polymer Science and Engineering, College of Chemistry and Chemical Engineering, Nanjing University, Nanjing, P.R. China; 3 Department of Pharmaceutical Analysis, China Pharmaceutical University, Nanjing, P.R. China; 4 Key Laboratory of Analytical Chemistry for Life Science, School of Chemistry and Chemical Engineering, Nanjing University, Nanjing, P.R. China; 5 Department of General Surgery, Drum-Tower Hospital, Medical School of Nanjing University, Nanjing, P.R. China; Faculty of Medicine, University of Porto, Portugal

## Abstract

**Aims:**

The matrix metalloproteinase (MMP) 2/9, also known as collagenases IV and gelatinases A/B, play a key role in cancer invasion and metastasis. However, the clinical trials of the MMP inhibitors (MMPIs) ended up with disappointing results. In this paper, we synthesized a gelatinase-responsive copolymer (mPEG-PCL) by inserting a gelatinase cleavable peptide (PVGLIG) between mPEG and PCL blocks of mPEG-PCL for anticancer drug delivery to make use of MMP2/9 as an intelligent target for drug delivery.

**Materials and Methods:**

mPEG-pep-PCL copolymer was synthesized via ring-opening copolymerization and double-amidation. To evaluate whether Nanoparticles (NPs) prepared from this copolymer are superior to NPs prepared from mPEG-PCL, NPs prepared from mPEG-PCL copolymer were used as positive control. Docetaxel-loading NPs using mPEG-pep-PCL and mPEG-PCL were prepared by nano-precipitation method, mentioned as Gel-NPs and Con-NPs, respectively. The morphologic changes of the NPs after treatment with gelatinases were observed macroscopically by spectrophotometer and microscopically by transmission electron microscopy (TEM) and atomic force microscopy (AFM). The cellular uptake amount and cytotoxicity of Gel-NPs and Con-NPs, respectively, in cell lines with different levels of gelatinase expression were studied. Moreover, the cytotoxicity study on the primary cancer cells isolated from pericardial fluids from a patient with late-stage lung cancer was conducted.

**Results:**

The Gel-NPs aggregated in response to gelatinases, which was confirmed macroscopically and microscopically. The cellular uptake amount of Gel-NPs was correlated with the level of gelatinases. The *in vitro* antitumor effect of Gel-NPs was also correlated with the level of gelatinases and was superior to Taxotere (commercially available docetaxel) as well as the Con-NPs. The cytotoxicity study on the primary lung cancer cells also confirmed the effectiveness of Gel-NPs.

**Conclusion:**

The results in this study preliminarily demonstrated the effectiveness of gelatinase-responsive targeting strategy and the prospect of this intelligent nano-drug delivery system though further studies are needed.

## Introduction

The latest decade witnessed the rapid development of nanoparticulate drug carriers as one of the most promising and effective modalities for targeted cancer therapy [1], [2]. The most striking feature of nanoparticulate carrier is to deliver drug specifically to the tumor tissue, in order to maximize the effectiveness and minimize the side effects of anti-cancer drugs. The targeting strategies originally included passive targeting strategies (the EPR effect [3]–[4]) and active targeting strategies (the use of ligands such as antibodies [5]–[7]). Recently, stimuli-responsive targeting has been emerging as the most promising strategy, where the delivery system becomes a participant, rather than merely a passive vehicle, in the optimization of therapy [8], [9]. As to stimuli-responsive targeting, the vehicle will aggregate or collapse according to a certain trigger, leading to drug release or uptake of drugs by cancer cells. The widely used triggers include physical factors such as heat, magnet or pH values (thermo-responsive [10], magnetic-responsive [11] and pH-responsive targeting [12]–[14], respectively). The thermo and magnetic targeting strategies require additional devices (thermotherapy machine or magnetic field), so the location of tumor should not be unidentified or disseminated. Consequently, these two strategies cannot be applied to disseminated tumors or undiscovered micro-metastatic tumors, the treatments of which are the most urgently needed in clinical practice. pH value is also used as a trigger because of the lower pH value in the tumor tissue. However, according to our latest findings, the pH-responsive strategy may suffer from pH-induced physiological drug resistance (PIPDR) thus failed to reach an adequate intracellular concentration [15]. Therefore, more exquisite triggers are highly required to realize the optimization of anticancer drugs.

The matrix metalloproteinase (MMP) family, which consists of at least 21 zinc-dependent endopeptidases, plays a key role in cancer invasion and metastasis [16], [17]. Among the various MMPs, MMP2/9, which are also known as collagenases IV and gelatinases A/B, have been reported to be the most important cancer-related MMPs. Beside their basic function of degrading the extracellular matrix (ECM), gelatinases also play a vital role in numerous malignant tumor behaviors, especially metastasis [18]–[20]. Furthermore, a number of clinical studies have revealed a definite correlation between gelatinases expression and poor outcomes of tumors [21]–[23]. Accordingly, MMP inhibitors (MMPIs) especially the MMPIs of MMP2/9 were expected to be an ideal category of anticancer agents since the 1970’s and a couple of MMPIs have entered Phase III clinical trials. However, all of these trials ended up unexpectedly with disappointing results. Some trials even terminated prematurely because patients in the MMPI group showed poorer survival than placebo-treated patients [18], [19]. The failures in the development of MMPIs indicate a more sophisticated function of MMPs in the development and metastasis of cancer. Recent studies revealed that MMPs may impact nearly every stage of cancer progression, including cancer growth, apoptosis, angiogenesis, invasion, metastasis and immune responses [18], [24]. Besides, the MMPs sometimes even play a protective role in tumor progression [25], [26]. As a result, merely inhibiting the function of MMPs will inevitably cause unpredictable consequences.

**Figure 1:**
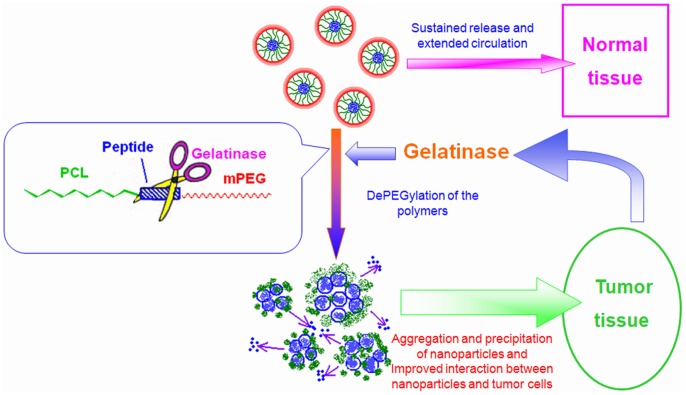
The gelatinase-stimuli strategy enhanced nanoparticles interactions with cancer cells in the tumor tissues.

Nevertheless, MMPs are undoubtedly important anticancer targets as their widespread expression and close relation to cancers. In this paper, we proposed that nanotechnology can make better use of MMPs as a promising target without bringing about undesirable side effects caused by MMPIs. Based on our previous studies on methoxy poly (ethylene glycol)–polycaprolactone (mPEG-PCL) copolymers [27], we modified the copolymer by inserting the optimal gelatinase cleavable peptide (PVGLIG) between mPEG and PCL segment (mPEG-Pep-PCL). Owing to the di-block structure and PEGylation, the nanoparticles (NPs) prepared from mPEG-Pep-PCL is originally characterized by prolonged circulating time and accumulation in the tumor site by EPR effect [3], [28], [29]. As gelatinases were extracellularly secreted into the tumor microenvironment, the mPEG-PCL conjugates will be cleaved at the certain site of the peptide. This dePEGylation process triggers the gathering of PCL blocks as well as the increased hydrophobicity of the aggregated NPs. Eventually, the dePEGylated NPs will interact with tumor cells more effectively, leading to increased cellular uptake of NPs into cancer cells and improved intracellular anticancer drug concentration (See Fig. 1.). According to this strategy, as the gelatinases work just as a specific trigger for drug accumulation, the expression of gelatinases will not be influenced and the side effects of MMPIs will not occur.

To confirm whether the characteristics of Gel-NPs changes in response to gelatinases and whether this change brings into improved antitumor efficiency, in this study, we loaded the mPEG-Pep-PCL copolymer with docetaxel, a widely used chemotherapeutic agent. The structure and gelatinase-responsive profile of mPEG-Pep-PCL NPs was studied by morphological observation, *in vitro* cytotoxicity evaluation and *in vitro* cellular uptake study. Taxotere (Commercially available docetaxel) as well as NPs prepared from mPEG-PCL was used as positive controls. Moreover, cytotoxicity study on cancer cells isolated from the pericardial fluids of a late stage lung cancer patient was also conducted to evaluate the effectiveness of this novel intelligent drug delivery system.

## Materials and Methods

### Ethics Statement

The study in this paper involving effusion samples collected from a 71 year old patient was conducted according to the principles expressed in the Declaration of Helsinki and had been approved by the Ethics Committee of Drum-Tower Hospital, Medical School of Nanjing University (DTH ERMH 46.10/210A/2011). Written informed consents were obtained from the patient.

### Materials

Methoxy-polyethyleneglycol-NHS (mPEG-NHS, Mn 5000, polydispersity≤1.08) was custom-synthesized by Beijing Jiankai Technology Co., Ltd. (Beijing, China). The MMP2/9-cleavable peptide (sequence: H2N-PVGLIG-COOH, which was designed according to the cleavage-site specificity study reported by Turk et al. [30]) was synthesized by Shanghai HD Biosciences Co., Ltd. (Shanghai, China). ε-caprolactone (ε-CL, Fluka, USA) and Dimethyl formamide (DMF) were purified by dehydrated over CaH_2_ at room temperature and distillated under reduced pressure. Docetaxel were kindly provided by Jiangsu Hengrui Medicine Company (Jiangsu, China) and Taxotere was purchased from Sanofi-Aventis (France), Coumarin-6, collagenase IV (Gibco, USA) 2,2'-(Ethylenedioxy)-diethylamine, di-tert-butyl dicarbonate, N-(3 -dimethylaminopropyl)- N'-ethylcarbodiimide hydrochloride (EDC×HCl), N- hydroxysuccinimide (NHS), 4-Dimethylaminopyridine (DMAP), RPMI 1640 and MTT (3-(4,5–dimethylthiazo l-2-yl)-2,5-diphenyltetrazolium bromide) were purchased from Sigma Chemical Company (USA). Cell Counting Kit-8 was from Dojindo, Kumamoto (Japan). All the other chemicals used were of the analytical grade available. Human embryo kidney cell 293T and human gastric cancer cell line BGC823 were purchased from Shanghai Institute of Cell Biology (Shanghai, China).

### Synthesis of mPEG-Pep-PCL and mPEG-PCL Copolymers

#### Synthesis of mPEG-Peptide conjugate

The mPEG-NHS (200 mg) and peptide (26 mg) were dissolved in dimethyl formamide (DMF) (3 mL) in the presence of triethylamine (3%) and stirred for 3 hrs at room temperature. After removing the unconjugated peptide by dialysis (MWCO 2000 Da) in deionized water, the purified PEG-Peptide conjugates were dehydrated for further use.

#### Synthesis and amination of PCL-COOH

A predetermined amount of ε-CL and L-leucine were added into a polymerization tube, and then the tube was vacuated, sealed off and put into an oil bath at 160°C for 24 hrs. The crude polymers were dissolved with dichloromethane (DCM) and precipitated into a large amount of cold ethyl ether to remove the un-reacted leucine, monomer and oligomer. The molecular weight of PCL-COOH was measured by gel permeation chromatography (GPC) (Waters 244, Milford, MA, USA) with 1 mL/min tetrahydrofuran (THF) as the eluent. Then the PCL-COOH was mixed with 1,8-Diamino-3,6-dioxaoctane, EDC, DMAP, NHS (the moral ratio was 10∶1, 5∶1, 7.5∶1 and 5∶1, respectively) and dissolved in DMF. The resulted solution was stirred in water bath at 37°C for 18 hrs. Then the resultant solution was precipitated into excess anhydrous ethanol to remove the impurities for three times. At last, the precipitation was washed with distilled water for several times and vacuum dried for further use.

#### Synthesis mPEG-Pep-PCL and mPEG-PCL

A solution of PCL- NH_2_ in DMF was added to a mixture of mPEG-Peptide conjugate, EDC and NHS. The reaction mixture was stirred for 24 hrs at 30°C. The crude mPEG-Peptide-PCL copolymer was purified by dialysis (MWCO13000) for 24 hrs, dehydrated to powder, stored at 4°C for further use. The mPEG-PCL di-block copolymers were synthesised by a ring opening copolymerization as we previously described [27]. ^1^H NMR (CDCl_3_) measured on a BrukerMSL-300 spectrometer was applied to determine the composition of the above two copolymers.

### Preparation of Gel-Doc-NPs and Con-Doc-NPs

Doctaxel (Doc)-loaded NPs (Gel-Doc-NPs) were prepared by modified nano-precipitation method. Briefly, 20 mg of copolymer and certain amount of Doc were dissolved in 200 µL acetone. Then the mixture was added into 6 mL deionized water quickly. The NPs formed and the solution turned into bluish immediately. The remaining acetone was removed by rotary vacuum evaporation and the resulted solution was filtered to remove non-incorporated Doc. Doc-loaded mPEG-PCL NPs (Con-Doc-NPs) were prepared in the same way. Blank NPs were produced in the same manner without adding Doc. Finally, all the nanoparticle suspensions were freeze dried with 4% F68 for further use.

### Drug Loading Content and Encapsulation Efficiency

To determine the drug-loading content, the freeze-dried powder of the NPs was dissolved in acetonitrile. Doc concentration in the resulted solution was then determined by the ultraviolet absorption at the wavelength of 230 nm, a strong absorption band of Doc with reference to a calibration curve on a Shimadzu UV3100 spectrophotometer (Shimadzu, Japan). Then the total amount of the drug in the NPs could be calculated.

Drug-loading content and encapsulation efficiency were obtained by the following equations [27]:
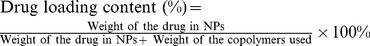
(1)

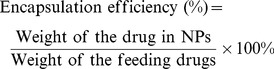
(2)


### Particle Size Measurement of NPs

The particle size and polydispersity of Gel-Doc-NPs and Con-Doc-NPs were measured by dynamic light scattering (DLS) (Brookhaven Instruments Corporation, USA). The values were the average of triplicate measurements for a single sample.

### Macroscopic Changes of NPs in Response to Gelatinase

Gel-Doc-NPs and Con-Doc-NPs were incubated with Hank’s solution containing gelatinase (Collagenase IV) at different concentrations at 37°C for 24 hrs. And the transparency of the solutions was observed by naked eyes as well as measured by a 721–100 visible spectrophotometer. Shanghai Precision & Scientific Instrument co. Ltd). The absorbance of 450 nm was measured.

### Microscopic Morphological Changes of NPs in Response to Gelatinase

The microscopic morphology examination of the Gel-Doc-NPs (incubated with or without gelatinases) was conducted using JEM-100S (Japan) transmission electron microscope (TEM). For TEM, One drop of NP suspension was placed on a copper grid covered with nitrocellulose membrane and air-dried before observation.

Atomic force microscope (AFM) (SPI3800, Seiko Instruments, Japan) was used to study the surface morphology of NPs in a greater detail. One drop of properly diluted NP suspension was placed on the surface of a clean silicon wafer and dried under nitrogen flow at room temperature. The AFM observation was performed with a 20 µm scanner in a tapping mode.

### Cytotoxicity of NPs to Cell Lines Expressing Different Level of Gelatinases

The Human embryo kidney cell 293T was of high level gelatinase expression and human gastric cancer cell line BGC823 was of comparatively low level of gelatinase expression according to gelatin zymography (Details in Data S1).

Cytotoxicity of Taxotere, Gel-Doc-NPs and Con-Doc-NPs against 293T as well as BGC-823 was assessed by MTT assay. Cells were seeded in 96 well plates with a density around 5000 cells/well. The cells were exposed to various concentrations of Taxotere, Gel-Doc-NPs and Con-Doc-NPs at 37°C. After co-incubation for 48 hrs, 20 µL of 5 mg/mL MTT solution was added to each well and the plate was incubated for another 4 hrs, allowing the viable cells to transform the yellow MTT into dark-blue formazan crystals, which were dissolved in 200 µL of dimethyl sulphoxide (DMSO). The optical density (OD) of each well was measured by an ELISA reader (ELX800 Biotek, USA) using test and reference wavelengths of 490 nm and 630 nm, respectively. Cell viability was determined by the following formula [27]:
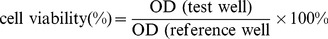
(3)


All the results obtained from MTT assays were confirmed by repeating the experiment on at least three independent occasions and testing in triplicate each time.

### *In vitro* Cellular Uptake Assays

The cellular uptake studies were taken according to our previous experiences [27]. 293T and BGC-823 cells were seeded onto the glass covers placed in a 6-well plate with RPMI 1640 supplemented with 10% calf serum at the density of 5×10^4^ cells per well. After incubation at 37°C in a humidified atmosphere with 5% CO_2_ for 24 hrs, cells were exposed to medium containing coumarin-6 loaded Gel-NPs (12.5 µg/mL). After incubation for 8 hrs, each glass cover was washed 3–4 times with PBS at 4 and 37°C respectively. Then the cells were observed under fluorescencemicroscopy (Olympus BX-51, wide band Blue excitation, exciter filter BP450-480, dichroic beamsplitterDM500, barrier filter BA515).

### The Antitumor Effect of NPs on Primary Cancer Cells Isolated from Pericardial Fluid

Effusion samples were collected from a 71 year old male patient with metastatic non-small cell lung cancer with pericardial effusion at the Comprehensive Cancer Center of Drum-Tower Hospital. The experimental application was approved by the Ethics Committee of Drum-Tower Hospital and written informed consents were obtained from the patient. Tumor cells were isolated from the pericardial fluid of the patient. Fresh effusions drained from patient were transferred to 50 mL tubes. Cells were prepared by centrifuging at 1500 rpm for 15 min followed by Ficoll-Hypaque (specific gravity 1.077, Pharmacia) density centrifugation. Erythrocytes in the pellet were lysed by mixing each 5 mL of the effusion suspension with 40 mL erythrocyte lysis buffer (155 mmol/L NH_4_Cl, 10 mmol/L NaHCO_3_, 1 mmol/L EDTA) and incubating at room temperature for 30 min. The number and viability of cells were determined by trypan blue dye and cytological examination. The final cell pellets were washed twice with PBS and prepared for cytotoxicity assay. Then cells were seeded in 96-well plates at 5×10^4^ cells/well and treated with various concentrations of Taxotere, Con-NPs and Gel-NPs in at least three replicate wells and left contact for 72 hrs. WST-8 reagent (10 µL) from Cell Counting Kit-8 was added to each well, and the cells were further incubated at 37°C for another 4 hrs. The optical density (OD) of each well was measured by an ELISA reader (ELX800 Biotek, USA) using test and reference wavelengths of 460 nm. Cell viability was determined by formula (3):

### Statistical Analysis

Statistical analyses of data were done using Student’s t test. The data are listed as mean±SD, and values of *P*<0.05 were accepted as a statistically significant difference.

## Results

### Preparation of mPEG-Pep-PCL Copolymer

**Figure 2:**
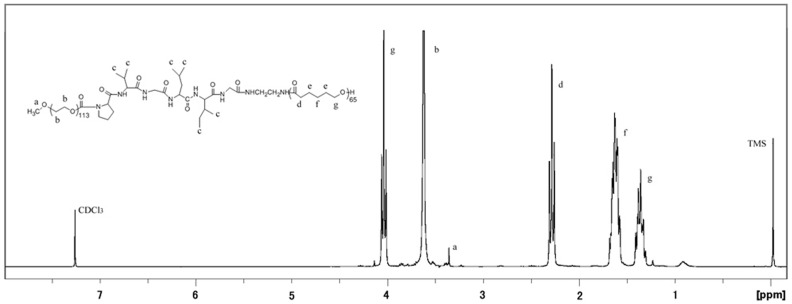
^1^H nuclear magnetic resonance spectra (300 MHz, 25°C) of PEG-Pep-PCL in CDCl3.

The mPEG-Pep-PCL copolymers were synthesized as described in the methods. Fig. 2. shows the ^1^H NMR spectra of mPEG-Pep-PCL copolymers in CDCl_3_, indicating the peptide was successfully conjugated into the copolymer. (Details in Fig. S1.)

The mole ratio of hydrophilic block to hydrophobic block (mPEG/PCL) in mPEG-Pep-PCL copolymer was about 1.771 based on the integral ratio of -CH2-O- (4.044 ppm) in PCL segment to -CH2–CH2-O (3.623 ppm) in mPEG segment from ^1^H NMR measurement. Thus the number-average molecular weight (Mn) of resulting mPEG-Pep-PCL copolymer was determined to be approximately13900 (Fig. 2.).

### The Hollow Structure of Gel-NPs

As to Table 1, the diameter of Gel-Doc-NPs was about 127.9 nm measured by DLS and the polydispersity index of Gel-Doc-NPs was 0.122, whereas the diameter of Con-Doc-NPs was about 89.6 nm and its polydispersity index was 0.089. The diameter of Gel-Doc-NPs was significantly larger than Con-Doc-NPs. (*P*<0.05, Table 1.).

**Table 1:** Characterization of the Gel-Doc-NPs and Con-Doc-NPs.

Nanoparticles	Diameter (nm)	Polydispersity index	Drug loading content (%)	Drug encapsulation efficiency (%)
Gel-Doc-NP	127.9±9.9	0.122±0.12	0.216	71.3
Con-Doc-NP	89.6*±7.0	0.089±0.02	0.184	68.4

* *P*<0.05.

**Figure 3:**
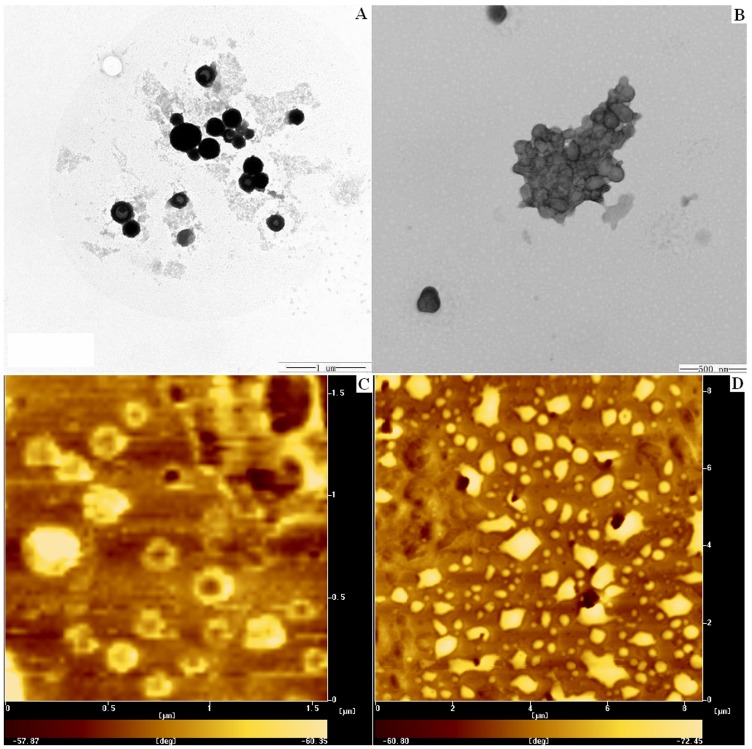
TEM (3-A and 3-B) and AFM (3-C and 3-D) images of Gel-NPs before (3-A and 3-C) and after (3-B and 3-D) incubation with CollagenaseIV.

From the images of TEM and AFM (Fig. 3A and Fig. 3C.), it could be observed that most of the NPs exhibited a spherical and hollow structure with the size of 100–150 nm in diameter, which coincided with the data from DLS.

### Macroscopic Morphologic Changes of NPs in Response to Gelatinase

**Figure 4:**
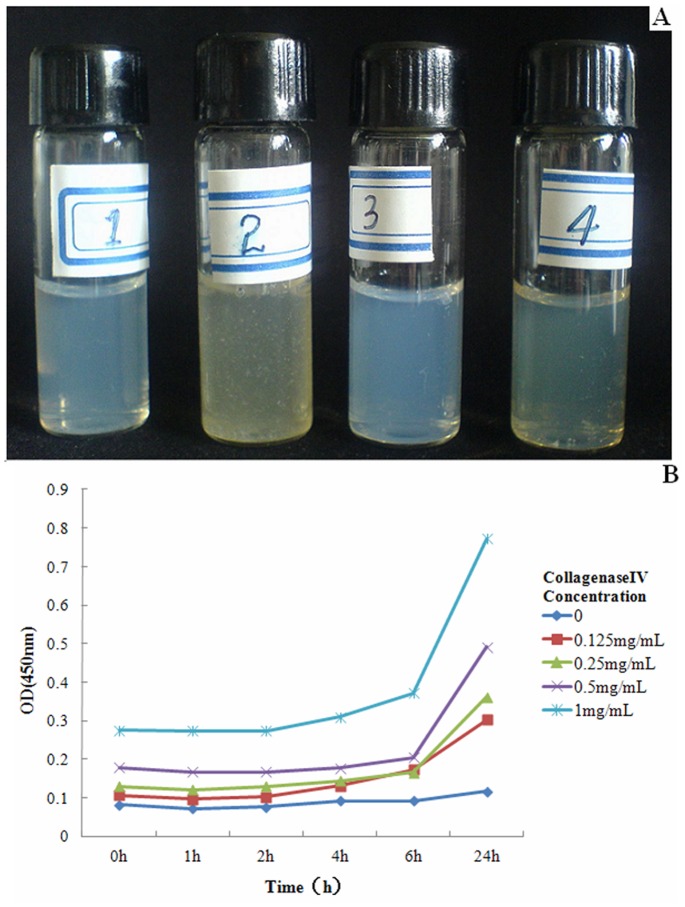
Gel-NPs and Con-NPs were incubated with Hank’s solution, Hank’s solution with gelatinase (Collagenase IV) at several concentrations at 37°C for 24 hrs. For 4-A, sample 1, refers to Gel-NPs without CollagenaseIV, sample 2 refers to Gel-NPs with CollagenaseIV, sample 3 refers to Con-NPs without CollagenaseIV and sample 4 refers to Con-NPs with CollagenaseIV, respectively. After incubating for 24 hrs, precipitation was only found in Sample4 (Gel-NPs with CollagenaseIV). 4-B showed the relationship between absorbance (indicating the amount of precipitation) and CollagenaseIV Concentration.

As to Fig. 4-A after incubation with gelatinases for 24 hrs, precipitation occurred only in Gel-NP suspension, indicating the aggregation of NPs due to the breakup of mPEG-Pep-PCL. However, Con-NP suspension incubated with gelatinase kept transparent. Moreover, both of the NP suspensions incubated with gelatinase-free Hank’s solution also kept transparent after 24 hrs.

The aggregation of NPs was also measured by visible spectrophotometer as the breakdown of NPs increased the turbidity of the liquid. As to Fig. 4-B, the turbidity of the liquid initially increased after 6 hrs of incubation and the absorbance of the suspension was also proportional to the concentration of gelatinases.

### Microscopic Morphological Changes of NPs in Response to Gelatinase

As shown in both TEM and AFM images (Fig. 3-B and Fig. 3-D), after incubation with gelatinase for 24 hrs, the aggregation of Gel-NPs was obvious.

### *In vitro* Cellular Uptake by Cells Expressing Different Level of Gelatinase

**Figure 5:**
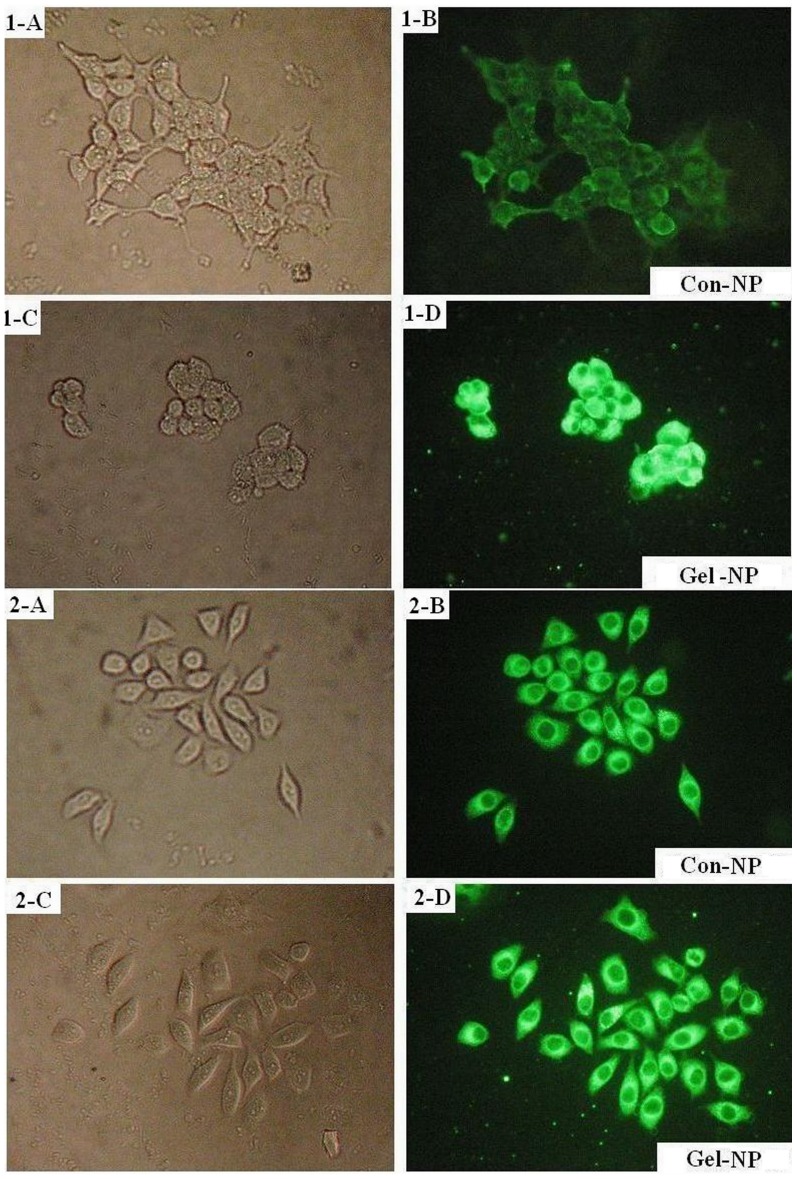
Fluorescence microscopic images of BGC-823 (gelatinase low expressing) and 293T (gelatinase-high expressing) cells after 8 hrs’ incubation with coumarin-6-loaded Con-NPs and Gel-NPs. The uptake of coumarin-6-loaded NPs in cells was visualized by FITC filter (magnification  = 200×). Fig. 5-1-A and 5-1-B: the optical microscopic and fluorescence microscopic images of BGC-823 cells incubated with coumarin-6 loaded Con-NPs for 8 hrs. Fig. 5-1-C and 5-1-D: the optical microscopic and fluorescence microscopic images of BGC-823 cells incubated with coumarin-6 loaded Gel-NPs for 8 hrs. Fig. 5-2-A and 5-2-B: the optical microscopic and fluorescence microscopic images of 293T cells incubated with coumarin-6 loaded Con-NPs for 8 hrs. Fig. 5-2-C and 5-2-D: the optical microscopic and fluorescence microscopic images of 293T cells incubated with coumarin-6 loaded Gel-NPs for 8 hrs.

Most cancer cells express gelatinases [20], [31]. As a result, in this study, the BGC-823 cell line, which expressed comparatively low level of gelatinase (significantly lower than 293T, *P*<0.05, see Fig. S2.) was selected as a negative control. The cellular uptake of NPs by cells is presented in Fig. 5. For both BGC-823 and 293T, the fluorescence intensities of Con-NPs were weaker than those of Gel-NPs. (5-1-B vs 5-1-D and 5-2-B vs 5-2-D). However, the difference of fluorescence intensity between Gel-NPs and Con-NPs was much more prominent in 293T cells than in BGC-823 cells (5-1-D vs 5-2-D). Furthermore, in the Gel-NP group (5-1-D and 5-2-D), fluorescent dots could be found in the background and there were more dots in the background of 293T cells incubated with Gel-NPs.

### Cytotoxicity of NPs against Cell Lines Expressing Different Level of Gelatinase

**Figure 6:**
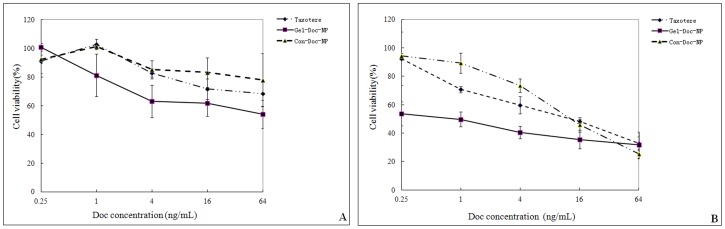
Cytotoxicity of NPs to cell lines expressing different level of gelatinase. The in vitro cytotoxicity of Taxotere, Gel-Doc-NPs and Con-Doc-NPs to BGC-823 (gelatinase low expressing) and 293T (gelatinase-high expressing) cells at different concentrations. The cytotoxicity was determined by the viabilities of cells. The data were presented as mean±SD.

Fig. 6-A (BGC-823) and Fig. 6-B (293T) presented the cytotoxicities of commercially available Doc (Taxotere), Gel-Doc-NPs and Con-Doc-NPs on BGC-823 cell line and 293T cell line. In both cell lines, the Gel-Doc-NPs exhibited a more prominent cytotoxicity compared with Taxotere as well as Con-Doc-NPs. However, the superiority of Gel-Doc-NPs was more notable in the group of 293 T.

### The Antitumor Effect of NPs on Primary Cancer Cells Isolated from Pericardial Fluid

**Figure 7:**
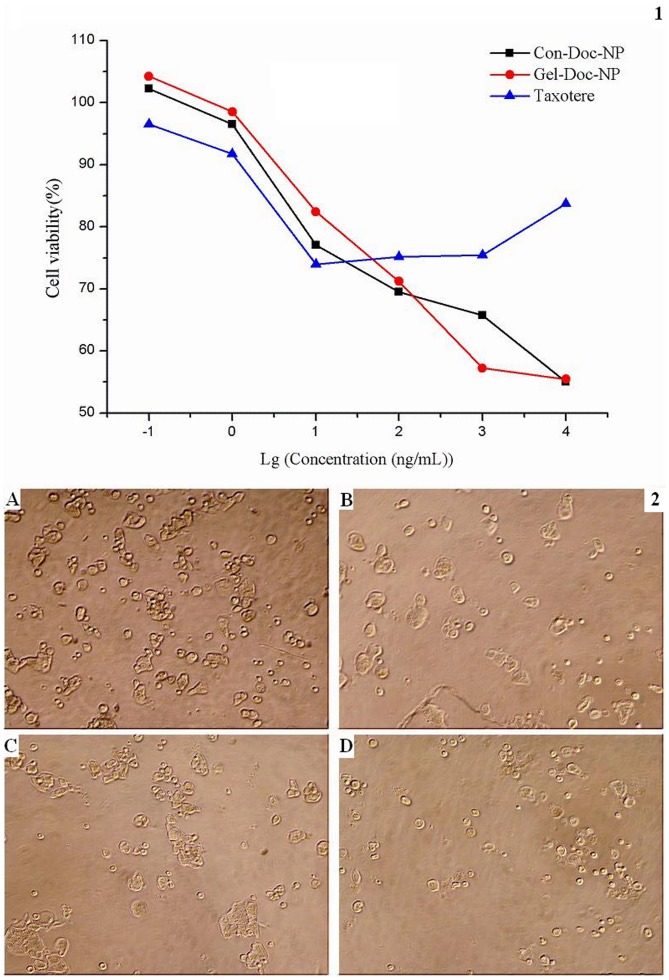
The antitumor effect of NPs on primary cancer cells isolated from pericardial fluid. Fig. 7-1 The in vitro cytotoxicity of Taxotere, Gel-Doc-NPs and Con-Doc-NPs to primary cells isolated from malignant pericardial fluid at different concentrations. The cytotoxicity was determined by the viabilities of cells. Fig. 7-2 Images of primary cells isolated from malignant pericardial fluid after no treatment(A), treatment with Taxotere(B), treatment with Con-Doc-NPs(C) and treatment with Gel-Doc-NPs(D). (magnification  = 100×).

The primary cancer cells isolated from the pericardial fluid of a late-stage lung cancer patient were treated with Taxotere, Con-Doc-NPs and Gel-Doc-NPs, respectively. As to Fig. 7-1, Taxotere could only inhibit the growth of cells not more than 28% even at the highest concentration. The cytotoxicity of Gel-Doc-NPs and Con-Doc-NPs were close to each other. However, there was a better linear relationship between Doc concentration and viabilities of cells in Gel-Doc-NPs group.

## Discussion

In this paper, we designed a new gelatinase-responsive nanoscale drug carrier and preliminarily demonstrated that in the presence of gelatinase, the structure of gelatinase-responsive NPs collapsed and the cellular uptake of drugs increased, leading to enhanced cytotoxicity of NPs.

Enzyme-responsive targeting is a newly developed and promising strategy in the area of stimuli-responsive drug delivery [8], [9], [32]–[35]. Compared to other triggers such as heat, pH value, magnet, etc., gelatinases show their superiority in the following aspects: firstly, gelatinases are specifically and highly expressed in a variety of tumors and correlate with tumor invasion as well as poor prognosis [21], [36]–[40]. As a result, gelatinases-responsive strategy could target the most invasive cancer cells. Secondly, gelatinase-responsive targeting is intelligent because it works without any additional devices thus can target microscopic, multiple or disseminated tumor lesions, even if the lesions are not clinically detectable. Thirdly, though gelatinases are synthesized intracellularly, active gelatinases are secreted and work extracellularly [16], [24], ensuring the aggregation of the NPs when they circulating to the tumor microenvironment.

Gelatinases are ideal triggers also due to their wide substrate spectrum. Gelatinases cleave native collagen IV at a specific Gly-Leu or Gly-lle bond. However, it is widely reported that gelatinases have a broader specificity which includes cleavages at Gly-Val, Gly-Phe and Gly-Ala, etc., regardless of the secondary or tertiary structure of peptides or proteins [41]. In other words, short peptides with a certain sequence (cleavage-site motif) can also be cleaved by gelatinases. In this study, we chose the sequence PVGLIG from the reported pooled sequencing of peptide library mixtures by cleavage-site specificity study [30] and successfully inserted the peptide between mPEG and PCL copolymers as a “junction”.(Fig. 2.).

In order to explore whether the peptide junction would breakup in response to gelatinases and whether this process would lead to the collapse and aggregation of NPs, we firstly studied the morphological changes of the NP suspension macroscopically. As shown in Fig. 4., Gel-NP suspension turned muddy after incubation with gelatinase while Con-NP suspension kept transparent, indicating that the gelatinase could trigger the collapse of Gel-NPs only. Further microscopic studies by TEM and AFM clearly showed the collapse and aggregation of NPs.

For cellular study, we selected the cells expressing different levels of gelatinases because there are few cancer cell lines that do not express gelatinase at all. As to the gelatin zymography, BGC-823 cells were identified to be of comparatively low gelatinase expression and 293T were found to be of high gelatinase expression. Then we studied the cellular uptake and cytotoxicity of Con-NPs and Gel-NPs in the two cell lines.

In cellular uptake studies, the fluorescence intensities reflected the amount of NPs internalized by the cells. It can be found that in both BGC-823 and 293T cells, there were more Gel-NPs inside the cells. And the difference of fluorescence intensities between Gel-NPs group and Con-NPs group was more obvious in high gelatinase expressing cell lines. This result showed that gelatinases trigger the internalization of NPs. Besides, on the background of the images of Gel-NPs, some fluorescent dots can be found. These dots were probably the aggregated Gel-NPs because firstly, coumarin-6 is insoluble in water and secondly, these dots did not appear in the images of Con-NPs and last but not least, there were more dots in the image of 293T, which express higher level of gelatinases than BGC-823.

Similar to cellular uptake studies, cytotoxicity study also revealed the best antitumor effect in the group of Gel-NPs. Gel-Doc-NPs exhibited better cytotoxicity than Con-Doc-NPs and Taxotere in both 293T and BGC-823 cell lines. It can be found that the superiority of Gel-Doc-NPs was more obvious in 293T (highly gelatinase expressing) cells, which is in accordance with the results got from cellular uptake studies.

We also preliminarily tested the effectiveness of NPs on primary cancer cells isolated from pericardial fluid. As to Fig. 7, Taxotere failed to inhibit the growth of cancer cells effectively (Max inhibition rate was 28%). Gel-Doc-NPs and Con-Doc-NPs showed similar antitumor efficiency. The concentration-viability curve of Gel-Doc-NPs was linear while that of Con-Doc-NPs present S like shape, indicating that the cancer cells may be more sensitive to Gel-Doc-NPs.

The patient suffered from stage IV non-small cell lung cancer and underwent several cycles of systemic and local chemotherapy (docetaxel or paclitaxel was not used) before the pericardial fluid occurred. Unfortunately, the patient died 3 days after the collection of the pericardial fluid and we failed to get enough fluid for the detection of gelatinases in the pericardial fluid or to repeat the cytotoxicity study. However, gelatinases have been found to be significantly and specifically elevated in a number of malignant effusions including pericardial effusions. [17], [42] As a result, the superiority of Gel-NPs is probably owing to the gelatinase-responsive strategy.

In addition to the aggregation of NPs in response to gelatinase, some other advantages of the Gel-NPs were also noticed. Firstly, Gel-NPs make full use of PEGylation and avoid its shortcomings. PEGylation has been widely used in the development of drug delivery system because it can prolong blood circulation, increase tumor accumulation, reduce serum protein adherence and avoid the uptake by the reticuloendothelial systems (RES) [28], [29]. However, recent findings revealed that the introduction of PEG chains on the surface of copolymers decreased endocytosis of NPs into cells [39], [43]. This dilemma can be solved by gelatinase-responsive strategy in that when Gel-NPs accumulate at tumor tissue, gelatinases trigger dePEGylation of NPs, enhancing the endocytosis of NPs. This also partially explains why in cellular uptake studies, more Gel-NPs entered cancer cells than Con-NPs.

Secondly, the Gel-NPs exhibited a hollow structure. This hollow structure increased the drug loading content and encapsulation efficiency of NPs. The hollow structure also make the NPs larger, however, it is reported that EPR effect exists in NPs with size smaller than 200 nm [44].As a result, the Gel-NPs are also capable of efficiently accumulating in tumor tissue because of its diameter smaller than 200 nm and their PEG shell.

Another feature of the NPs is that PEG and PCL are all copolymers that approved by FDA for intravenous use [45]. Thus the mPEG-Pep-PCL copolymer didn’t contain any other materials whose biocompatibilities are under suspicion, which make the NPs potentially clinically applicable.

This study preliminarily verified the construction, gelatinase-responsive characteristics and the superior *in vitro* antitumor effect of Gel-NPs. However, to comprehensively evaluate this new drug delivery system, further study is required to study the *in vivo* antitumor effect of Gel-NPs and its relation to the expression of gelatinases; the *in vivo* side effects of this -NP should also be assessed. We are convinced that this gelatinases-responsive drug vehicle presented here have great potential as an intelligent drug delivery strategy and merit further and more detailed studies.

### Conclusions

In this paper, an intelligent drug delivery system based on gelatinase-responsive strategy and the biocompatible copolymer PEG and PCL was developed. We firstly confirmed that the structure of the mPEG-Pep-PCL copolymer was the same as what we designed. Then the morphological changes of the NPs exhibited their gelatinase sensitivity. *In vitro* studies on cancer cell lines as well as primary cancer cells confirmed that the docetaxel-loaded gelatinase-responsive NPs exhibited more drastic antitumor effect over commercially available Docetaxel formulation (Taxotere) together with the NPs prepared from mPEG-PCL, and this superiority was more obvious in the cells expressing high level of gelatinases. Moreover, the gelatinase-responsive NPs presented a hollow structure, leading to higher drug content. The results from this study preliminarily exhibited the superiority and application potential of this gelatinase-responsive NPs. As gelatinases are specifically highly expressed in most of the tumors and are closely related to the invasion and metastasis of tumors, gelatinase-targeting is of greater prospect in the treatment of metastatic or disseminated tumors, which are the main hurdles in clinical oncology.

## Supporting Information

Figure S1 **Synthesis scheme of PEG-Pep-PCL copolymers.** A represents PEG-Pep and B represents PCL-COOH. The ^1^H nuclear magnetic resonance spectra (300 MHz, 25°C) of PEG-Pep in CDCl_3_ and GPC chromatogram of PCL-COOH were also shown. Abbreviations: PCL, poly(ε-caprolactone); PEG, poly(ethylene glycol); Pep, gelatinase-responsive peptide (PVGLIG).
(TIF)

Figure S2 **Detection of gelatinases by gelatin zymography.** Following the Coomassie blue staining and color inverting, gelatinases activity was detected as a white zone on black background and quantified by densitometry. Figure S2-A Scanning images of the gelatin zymography for BGC-823 and 293T cells. Figure S2-B The expressions of gelatinase by BGC-823 and 293T cells using a semi-quantitative technique.
(TIF)

Data S1 **Supporting Materials and Methods.**
(DOC)
