# Supplementary figures and images for: Intelligently Targeted Drug Delivery and Enhanced Antitumor Effect by Gelatinase-Responsive Nanoparticles
Source: PLoS One. 2013 Jul 30;8(7):e69643. doi: 10.1371/journal.pone.0069643 (PMC3728361; doi:10.1371/journal.pone.0069643)

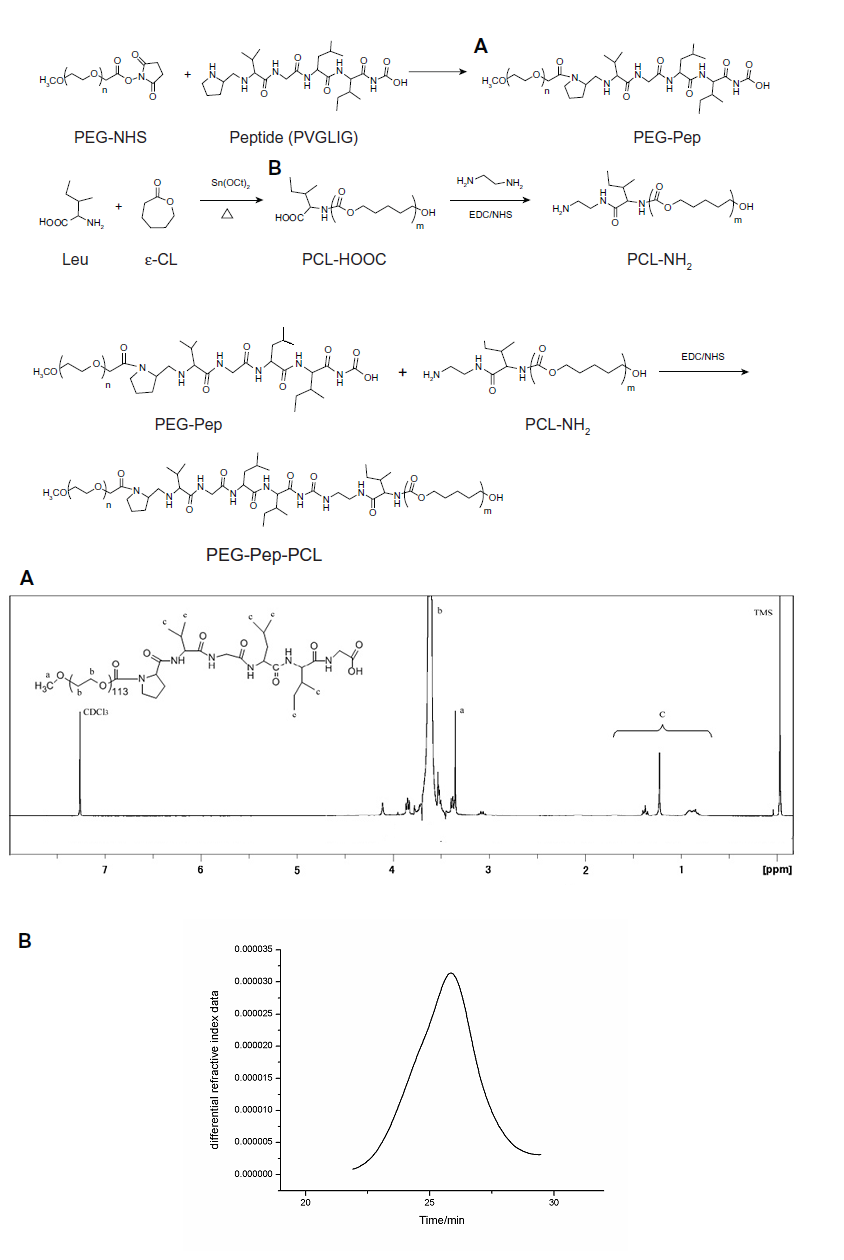

Supplement: Figure S1 — Synthesis scheme of PEG-Pep-PCL copolymers. A represents PEG-Pep and B represents PCL-COOH. The 1H nuclear magnetic resonance spectra (300 MHz, 25°C) of PEG-Pep in CDCl3 and GPC chromatogram of PCL-COOH were also shown. Abbreviations: PCL, poly(ε-caprolactone); PEG, poly(ethylene glycol); Pep, gelatinase-responsive peptide (PVGLIG). (TIF) [file pone.0069643.s001.tif]

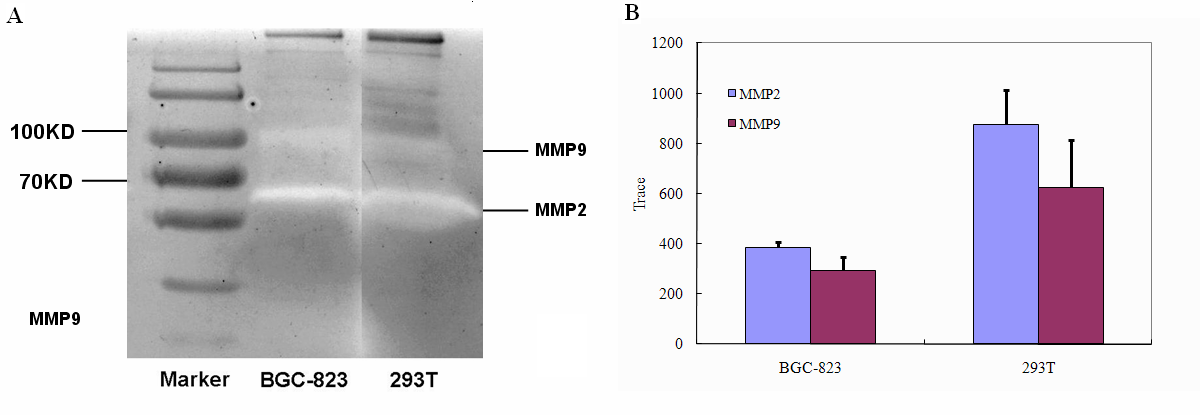

Supplement: Figure S2 — Detection of gelatinases by gelatin zymography. Following the Coomassie blue staining and color inverting, gelatinases activity was detected as a white zone on black background and quantified by densitometry. Figure S2-A Scanning images of the gelatin zymography for BGC-823 and 293T cells. Figure S2-B The expressions of gelatinase by BGC-823 and 293T cells using a semi-quantitative technique. (TIF) [file pone.0069643.s002.tif]
