# Supplementary material for: Intelligently Targeted Drug Delivery and Enhanced Antitumor Effect by Gelatinase-Responsive Nanoparticles
Source: PLoS One. 2013 Jul 30;8(7):e69643. doi: 10.1371/journal.pone.0069643 (PMC3728361; doi:10.1371/journal.pone.0069643)
Supplement: Data S1 — Supporting Materials and Methods. (DOC) [file pone.0069643.s003.doc]

Materials and methods

**Molecular weight and chemical composition analysis**

Molecular weight of PCL-COOH were determined by a gel permeation chromatography (GPC) (Waters 515 systems) equipped with Waters 1525 pump, Waters 410 differential refractometer, Waters utrahydrogel ™ linear columns. 50/50 water (double-distilled)/methanol (HPLC grade) containing 0.1 mol/L LiNO_3_ was used as eluent at a flow rate of 0.5 mL/min. The chemical structures of PEG-Pep-PCL were characterized on ^1^H NMR (Bruker DQX-300) using CDCl_3_ as the solvent for the sample. The composition of the samples was estimated from the ratio of the peak areas assigned to mPEG and CL blocks in the NMR spectrum. From the EG/CL molar ratio, the number-average molar mass (Mn) of copolymers was determined.

**Gelatin zymography**

Gelatin zymography was used to quantify the extracellular active gelatinase (MMP-2 and MMP-9) [1]. Briefly, 100 μL culture medium was subjected to 10% SDS-polyacrylamide gels co-polymerized with gelatin (final concentration of 1mg/mL). after electrophoresis, the gels were then incubated in 2.5% Triton X-100 for 2 hours and rinsed in distilled water. Gels were further incubated at 37°C for 36 hours in 200 mM NaCl, 5 mM CaCl2, 0.02% Brij-35, 50 mM Tris-HCl buffer (pH 8.8), 1 μM ZnCl2, then stained with 0.2% Coomassie brilliant blue R-250, and destained in 10% acetic acid and 30% methanol in H2O. After color inverting, gelatinolytic activity was detected as unstained bands on a blue background. Three independent experiments were performed.

**References**

1. Lane W, Dias S, Hattori K, Heissig B, Choy M, Rabbany S, et al. (2000) Stromal-derived factor 1-induced megakaryocyte migration and platelet production is dependent on matrix metalloproteinases. Blood 96: 4152-4159.
